# Hydropic Ear Disease: Correlation Between Audiovestibular Symptoms, Endolymphatic Hydrops and Blood-Labyrinth Barrier Impairment

**DOI:** 10.3389/fsurg.2021.758947

**Published:** 2021-11-04

**Authors:** Lisa M. H. de Pont, Josephine M. van Steekelenburg, Thijs O. Verhagen, Maartje Houben, Jelle J. Goeman, Berit M. Verbist, Mark A. van Buchem, Claire C. Bommeljé, Henk M. Blom, Sebastiaan Hammer

**Affiliations:** ^1^Department of Radiology, Haga Teaching Hospital, The Hague, Netherlands; ^2^Department of Radiology, Leiden University Medical Center, Leiden, Netherlands; ^3^Department of Otorhinolaryngology, Haga Teaching Hospital, The Hague, Netherlands; ^4^Department of Otorhinolaryngology, Leiden University Medical Center, Leiden, Netherlands; ^5^Department of Biomedical Data Sciences, Leiden University Medical Center, Leiden, Netherlands; ^6^Department of Otorhinolaryngology, Antwerp University Hospital, Antwerp, Belgium

**Keywords:** endolymphatic hydrops, blood-labyrinth barrier, magnetic resonance imaging, clinical features, audiovestibular function

## Abstract

**Research Objective:** To investigate the correlation between clinical features and MRI-confirmed endolymphatic hydrops (EH) and blood-labyrinth barrier (BLB) impairment.

**Study Design:** Retrospective cross-sectional study.

**Setting:** Vertigo referral center (Haga Teaching Hospital, The Hague, the Netherlands).

**Methods:** We retrospectively analyzed all patients that underwent 4 h-delayed Gd-enhanced 3D FLAIR MRI at our institution from February 2017 to March 2019. Perilymphatic enhancement and the degree of cochlear and vestibular hydrops were assessed. The signal intensity ratio (SIR) was calculated by region of interest analysis. Correlations between MRI findings and clinical features were evaluated.

**Results:** Two hundred and fifteen patients with MRI-proven endolymphatic hydrops (EH) were included (179 unilateral, 36 bilateral) with a mean age of 55.9 yrs and median disease duration of 4.3 yrs. Hydrops grade is significantly correlated with disease duration (*P* < 0.001), the severity of low- and high-frequency hearing loss (both *P* < 0.001), and the incidence of drop attacks (*P* = 0.001). Visually increased perilymphatic enhancement was present in 157 (87.7%) subjects with unilateral EH. SIR increases in correlation with hydrops grade (*P* < 0.001), but is not significantly correlated with the low or high Fletcher index (*P* = 0.344 and *P* = 0.178 respectively). No significant differences were found between the degree of EH or BLB impairment and vertigo, tinnitus or aural fullness.

**Conclusion:** The degree of EH positively correlates with disease duration, hearing loss and the incidence of drop attacks. The BLB is impaired in association with EH grade, but without clear contribution to the severity of audiovestibular symptoms.

## Introduction

Menière's disease (MD) is a refractory otologic disorder that predominantly manifests in adults between 40 and 60 years of age (1, 2). Its typical features are recurrent vertigo spells associated with fluctuating cochlear symptoms in the affected ear (3–6). The disease may affect one ear, albeit bilaterality has been reported in 2–73% of cases (7). MD is strongly associated with endolymphatic hydrops (EH): a distention of the endolymphatic space of the inner ear due to accumulation of endolymph fluid (8). Although a certain diagnosis of EH is reserved for post-mortem temporal bone histology, the Equilibrium committee of the AAO-HNS has provided clinical guidelines to aid the diagnosis of MD in living patients (5, 9).

The most striking features of MD are rotatory vertigo and hearing loss, which show great variability among patients with respect to onset, duration and severity (10, 11). Vertigo attacks can last from several minutes to hours, and are often incapacitating due to their unpredictability and impact on quality of life (12). In initial stages of the disease, hearing loss is typically reversible after each vertigo attack, but profound and permanent hearing loss ultimately develops (13). Additionally, patients may experience a myriad of symptoms consisting of (but not limited to) tinnitus, aural fullness, or a sudden fall without loss of consciousness known as drop attacks (14–16). Migraine and autoimmune diseases are common comorbidities, reported in up to 16 and 11% of patients respectively (17–21).

Despite their strong association, the relationship between EH and MD remains unclear. In the last decade, the application of delayed gadolinium (Gd)-enhanced MRI has provided novel insights in the clinical effect of EH (22–30). The degree of EH is reported to correspond with disease duration and the degree of sensorineural hearing loss in MD patients (27, 28, 31, 32). Contrarily, no correlation has emerged between EH and tinnitus, aural fullness, or the frequency and duration of vertigo attacks (23, 30). The absence of a clear correlation has led to the belief that other mechanisms beyond EH must play a role in the production of audiovestibular symptoms.

Recent literature has introduced novel MRI parameters that may help clarify the clinical-MRI inconsistency. Increased perilymph signal intensity (SI) at 4 h-delayed Gd-enhanced 3-dimensional fluid-attenuated inversion recovery (3D FLAIR) MRI is a frequent concomitant finding in patients with EH (32–36). The underlying pathophysiological process is assumed to be impairment of the blood-labyrinth barrier (BLB), leading to increased permeability and leakage of Gd from capillaries into the perilymphatic spaces (32, 37). The increased perilymphatic enhancement is well-visualized on 3D FLAIR, due to its high sensitivity to changes in T1-shortening induced by gadolinium-based contrast media (38). Previous studies investigating this FLAIR hyperintensity have reported that the concurrent finding of EH and BLB impairment slightly increases specificity for MD compared with EH alone (34, 35). The importance of this MRI parameter is further emphasized by the notion that BLB impairment may be the sole MRI correlate in a small percentage of MD patients without EH (35, 36). Furthermore, normalization of the FLAIR hyperintensity has been observed in coincidence with improved vestibulocochlear function in several inflammatory inner ear conditions (39–41). The above-mentioned findings suggest that BLB impairment may provide additional clinical information about HED and may potentially bring new insights to the individual severity of audiovestibular symptoms.

Extensive literature from the past two decades has demonstrated that virtually all patients with clinically overt MD show EH on MRI, for which different subjective EH grading systems have been used (34, 35, 42–44). However, probably the most important gain from hydrops MRI is the demonstration of EH in clinically atypical or non-classifiable cases according to the current AAO-HNS criteria (34, 45). These findings highlight the diagnostic relevance of MR imaging, particularly in the clinical setting where the current AAO-HNS criteria appear to be insufficient—e.g., subjects with isolated cochlear or vestibular symptoms. This concept has sparked aspirations to abandon the symptom-based classification and to adapt a diagnostic system that incorporates the MRI-objectivation of EH. In 2018, Gürkov et al. proposed the classification of Hydropic Ear Disease (HED): an imaging-based classification system that is based upon the demonstration of EH and that acknowledges the wide spectrum of audiovestibular symptoms associated with this pathological substrate (8, 46). However, the clinical utility of this system relies on the value of imaging-derived parameters in this clinical spectrum.

The objective of this study was therefore to investigate the correlation between clinical features and labyrinthine abnormalities in patients with MRI-proven hydropic ear disease, with emphasis on EH and BLB-impairment.

## Materials and Methods

### Patients

We retrospectively identified all patients who underwent 4 h-delayed Gd-enhanced 3D FLAIR MRI at our institution from February 2017 to March 2019. The commonest clinical indication for inner ear MRI was recurrent vertigo with fluctuating aural symptoms suspected of MD. Three-hundred eighty-nine patients were eligible for inclusion. Exclusion criteria were <18 years old (*N* = 1), previous middle or inner ear surgery (*N* = 17), prior ablative treatment of the ear with gentamicin injections (*N* = 10) or a technically inadequate MRI examination (*N* = 11). We also excluded patients with coexisting conditions that may cause fluctuating audiovestibular symptoms, such as otosclerosis (*N* = 2) and vestibular schwannoma (*N* = 2).

### MRI Protocol

Imaging examinations were carried out on a 3T MRI scanner (Magnetom Skyra; Siemens, Erlangen, Germany) with a 20-channel array head coil, 4 h after intravenous gadolinium administration (30 mL gadoterate meglumine, Dotarem; Guerbet, Aulnay-sous-Bois, France) (34). Patients were evaluated in the supine position with additional fixation between the patient's head and receiver coil to reduce motion artifacts. We acquired a 3D FLAIR sequence to differentiate endolymph from perilymph with the following parameters: FOV = 190 mm, section thickness 0.8 mm, TR = 6,000 ms, TE = 177 ms, number of excitations 1, TI 2,000 ms, flip angle 180°, matrix 384 × 384, bandwidth 213 Hz/pixel, turbo factor 28, voxel size 0.5 × 0.5 × 0.8 mm, resulting in an acquisition time of 14 min. High-resolution T2 sampling perfection with application-optimized contrasts by using different flip angle evolution (SPACE sequence; Siemens) images of the inner ear were obtained for anatomic reference of the entire labyrinthine fluid space. The scan parameters for the T2 SPACE sequence were as follows: FOV 160 mm, section thickness 0.5 mm, TR 1,400 ms, TE 155 ms, number of excitations 1, flip angle 120°, matrix 320 x 320, bandwidth 289 Hz/pixel, turbo factor 96, voxel size 0.5 × 0.5 × 0.5, and acquisition time 5 min.

### MRI Evaluation

#### Visual Assessment

Images were independently evaluated by two observers: one head and neck radiologist (SH) and one PhD student (LP) with, respectively, 4.5 and 3 years of experience in hydrops MRI, who were blinded to the clinical data. The presence of endolymphatic hydrops was graded on a 4-point scale for vestibular hydrops and a 3-point scale for cochlear hydrops, respectively (Table 1). The *summative EH score* was calculated as the sum of cochlear and vestibular EH grades per patient to reflect global EH severity, which could range from 0 to 5.

**Table 1:** Grading of endolymphatic hydrops on 4 h-delayed Gd-enhanced 3D FLAIR MRI.

**Grade**	**Vestibule**	**Cochlea**
0	Normal-sized saccule and utricle	No displacement of Reissner's membrane
1	The saccule is equal in size or larger than the utricle, but not confluent	Dilation of the scala media with partial obliteration of the scala vestibuli
2	Confluence of saccule and utricle that encompasses >50% of the vestibule	Complete obliteration of the scala vestibuli
3	Total effacement of the perilymphatic space in the vestibule	n.a.

*n.a. indicates not applicable*.

Post-contrast signal intensity of the perilymph was visually scored as normal (symmetrical) or increased (asymmetrical). Any discrepancy was resolved by consensus reading.

#### Quantitative Analysis

**Figure 1:**
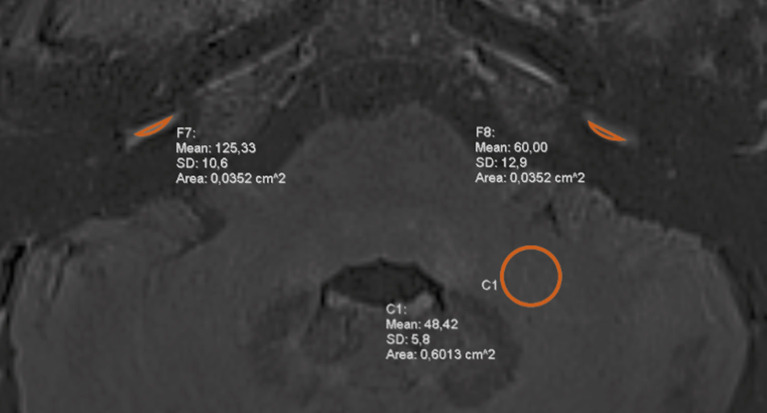
Signal intensity measurements at 4 h-delayed contrast-enhanced 3D FLAIR MRI. Symmetrical regions of interest (ROI) were drawn in the basal cochlear turn of each ear and a circular ROI was placed in the left middle cerebellar peduncle. The measurements were used to calculate the signal intensity ratio (SIR).

Quantitative measurements of the perilymph signal intensity were performed by one observer (LP) blinded to the clinical data. A freehand region of interest (ROI) was drawn on an axial section in the basal cochlear turn of both ears. In this region, quantification of perilymph enhancement was considered most reliable as the scala tympani is less affected by the morphological distortion from EH compared with other perilymphatic structures and this structure remains visible regardless of the degree of EH. In addition, the scala tympani visually appears the largest in the basal turn of the cochlea and is therefore easiest to contour in this region. An additional circular ROI of 0.6 mm^2^ was placed in the left middle cerebellar peduncle (Figure 1). The signal intensity ratio (SIR) of the basal cochlear turn to that of the middle cerebellar peduncle was calculated.

### Clinical Symptoms and Pure-Tone Audiometry

Clinical diagnoses were independently evaluated by 2 otorhinolaryngologists (HB and CB), who were blinded to the MRI results, according to the latest AAO-HNS criteria (5). Patients who did not fulfill the clinical criteria for probable or definite MD were assigned the clinical diagnosis *other disease*, which was considered an umbrella term for non-classifiable cases and patients with other vertigo-associated diseases (e.g., vestibular neuritis, vestibular migraine). Any discrepancy was resolved by consensus reading.

Information on the presence of audiovestibular symptoms and results from pure-tone audiometry (PTA) was collected from medical records. Age was defined as age at time of MRI. Unless otherwise specified, we calculated disease duration as the time elapsed from the first appearance of vertigo *or* hearing loss until MRI at our hospital. The frequency of vertigo attacks was calculated as the number of attacks per month. The duration of vertigo was divided into the following five categories: (1) <20 min; (2) 20 min to 12 h; (3) 12–24 h; (4) more than 24 h; and (5) variable duration. For hearing loss analysis, we selected the most recent PTA before or after MRI with a maximum time interval of 1 year. We documented hearing function in the form of low and high Fletcher indexes (the average hearing loss at the frequencies 0.5–1.0–2.0 kHz and 1.0–2.0–4.0 kHz, respectively).

### Statistical Analysis

Statistical analyses were performed using SPSS Statistics (version 24, IBM, Chicago, Illinois, USA). The level of significance was set at *P* < 0.05. Inter-observer agreement on the presence and grading of EH (3-point scale for the cochlea and 4-point scale for the vestibule) was estimated using Cohen's kappa (κ) coefficient. We considered a κ value >0.80 as excellent agreement. The Kolmogorov-Smirnov test was used to test the data for normal distribution. Data are presented as median [min-max] *or* mean ± standard deviation. Means and medians were compared between two independent groups by a Student *t* test and Mann-Whitney *U* test, respectively. The chi-square test was used to compare gender distribution and the prevalence of symptoms and comorbidities among groups with different hydrops grades. A Kruskall-Wallis *H* test was used to compare median EH scores and SIR across groups with different duration of vertigo attacks. The Wilcoxon rank-test was used to compare SIR and Fletcher indexes between both ears from unilateral EH patients. Correlation between (normally and not-normally distributed) continuous variables were assessed using the Pearson and Spearman correlation coefficients. Correlation between continuous and ordinal variables were assessed using the Jonckheere-Terpstra test of trend, which is a rank-based test that can be used to assess the significance of a trend in the data (e.g., as EH deteriorates, does hearing loss also deteriorate). Unless otherwise specified, correlation tests between MRI findings and clinical features were performed solely in patients with unilateral EH.

## Results

### Subject Characteristics

EH was observed in 215 patients (62.1%). Patient characteristics are listed in Table 2. The remaining 131 patients (37.9%) had no apparent EH and were excluded from further analysis. A summary of the clinical diagnoses of the included and excluded patients is provided in Table 3. Interobserver agreement for the clinical diagnosis was substantial (κ = 0.78).

**Table 2:** Clinical characteristics of patients with HED (*n* = 215).

Male/female, *n* (%)	101/114 (47/53)
Unilateral/bilateral EH, *n* (%)	179/36 (83/17)
Mean age, *y*	55.9 ± 0.9
Mean age of onset, *y*	47.5 ± 1.1
Vertigo, *n* (%)	212 (98.6)
Subjective hearing loss, *n* (%)	208 (96.7)
Tinnitus, *n* (%)	196 (91.2)
Aural fullness, *n* (%)	144 (67.0)
Median disease duration, *y*	4.25 [0.04–45.9]
Median duration hearing loss, *y*	5.1 [0.03–42.0]
Median duration vertigo, *y*	3.9 [0.04–45.9]
Migraine, *n* (%)	24 (11.2)
Autoimmune diseases, *n* (%)	14 (6.5)
Cardiovascular risk factors
High blood pressure, *n* (%)	37 (17.2)
Dyslipidemia, *n* (%)	20 (9.3)
Type II diabetes, *n* (%)	12 (5.6)
History of smoking, *n* (%)	15 (7.0)

*HED, Hydropic Ear Disease; EH, endolymphatic hydrops; N, number; Y, year*.

**Table 3:** Clinical diagnosis of the included and excluded patients according to the 2015 AAO-HNS criteria.

	**Subjects with EH (included)**	**Subjects without EH (excluded)**	***N* (%)**
Unilateral definite MD	174	6	180 (52.0%)
With contralateral asymptomatic ear	167	6	
With contralateral symptomatic ear	7	0	
Bilateral definite MD	17	4	21 (6.1%)
Unilateral probable MD	11	9	20 (5.8%)
With contralateral symptomatic ear	10	9	
With contralateral asymptomatic ear	1	0	
Bilateral probable MD	0	1	1 (0.3%)
Unilateral other disease	8	55	63 (18.2%)
Bilateral other disease	5	56	61 (17.6%)
Total	215	131	346 (100%)

*EH, endolymphatic hydrops; MD, Menière's disease; other disease, other vertigo-associated diseases and non-classifiable cases*.

### MRI Findings

#### Inter-observer Reliability

Interobserver reliability was excellent: κ = 0.88 for cochlear EH, κ = 0.95 for vestibular EH, κ = 0.83 for visual assessment of perilymph enhancement.

#### Endolymphatic Hydrops

All 215 patients demonstrated EH in their clinically affected ear, including 179 patients (83.3%) with unilateral EH (101 right, 78 left) and 36 patients with bilateral EH (16.7%). Concurrent vestibular and cochlear EH was most prevalent (144 patients, 67%). Isolated vestibular or cochlear hydrops was present in 53 (24.7%) and 18 (8.4%) patients, respectively. The degree of cochlear hydrops is strongly associated with the degree of vestibular hydrops, as revealed by the Spearman correlation coefficient (*r*_s_ = 0.553; *P* < 0.001).

Among the 36 patients with bilateral EH, 21 (58.3%) had symptoms in the contralateral hydropic ear and 15 (41.7%) were clinically silent on the contralateral side. Asymptomatic EH appeared more frequently in the vestibule (80%) than in the cochlea (26.7%).

#### Post-contrast Perilymph Signal Intensity

**Figure 2:**
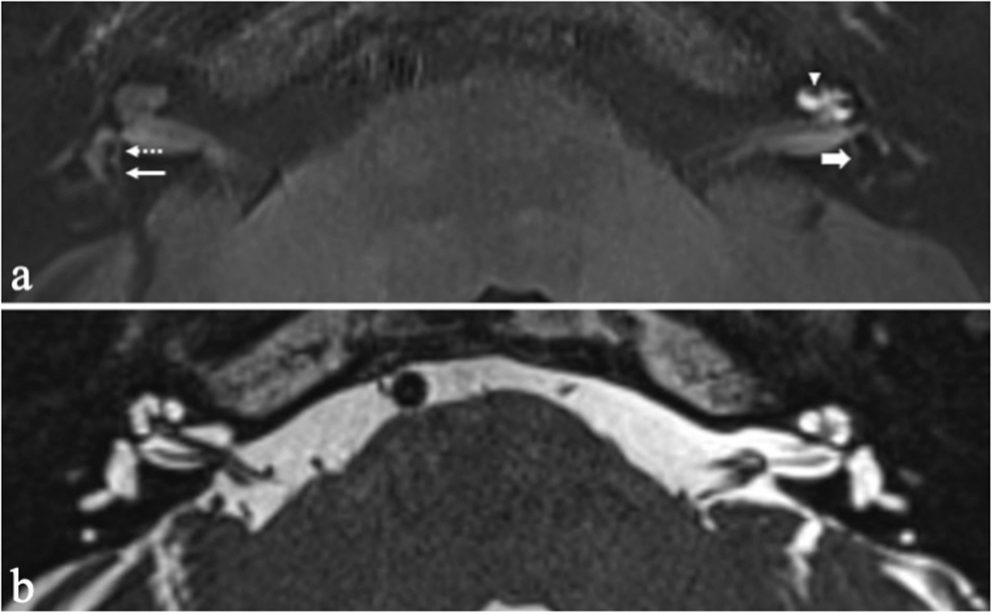
Axial 4 h-delayed Gd-enhanced 3D FLAIR MRI **(a)** and axial heavily T2 weighted sequence **(b)** in a patient with recurrent vertigo attacks and left-sided sensorineural hearing loss. The affected left ear demonstrates dilation of the scala media with complete obliteration of the scala vestibuli compatible with grade 2 cochlear hydrops (arrow head). The saccule and utricle in the left ear are confluent indicative of grade 2 vestibular hydrops (thick arrow). A thin rim of surrounding perilymph remains visible. Compare with a normal saccule (dashed arrow) and utricle (thin arrow) in the right ear. Also note the increased perilymphatic enhancement in the cochlea of the left ear, suggestive of BLB impairment.

**Figure 3:**
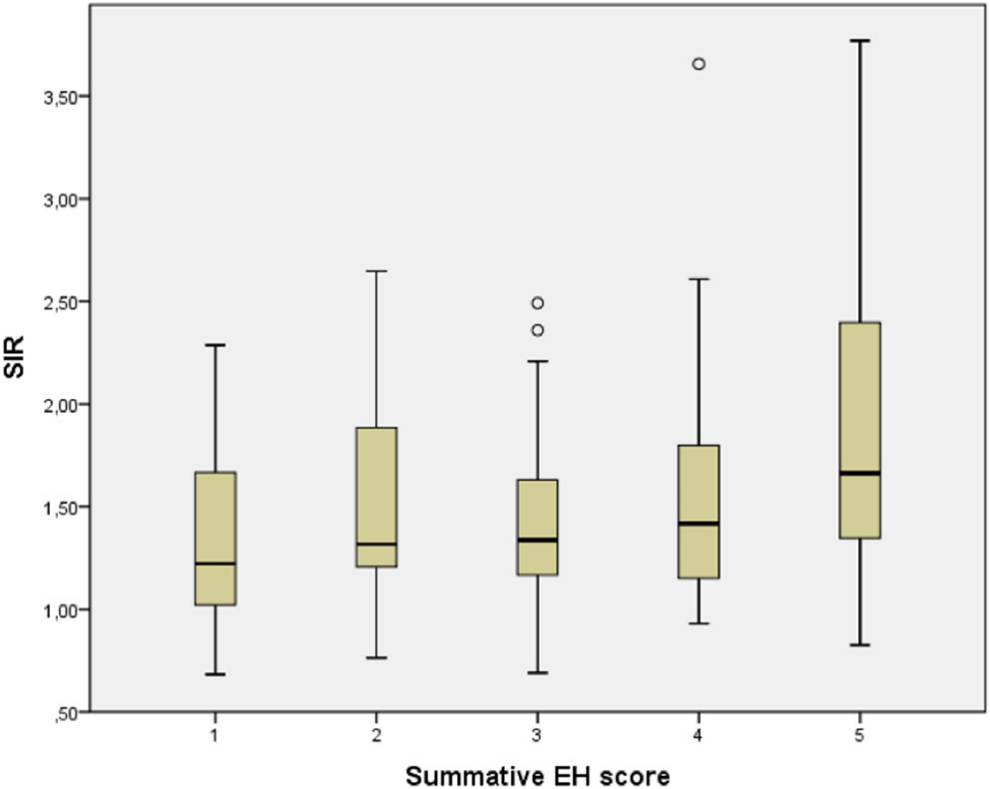
Correlation between signal intensity ratio (SIR) and summative endolymphatic hydrops (EH) score in unilateral hydropic ear disease (HED). A Jonckheere-Terpstra rank-test revealed a significant trend toward a higher median SIR among increasing EH score (*P* < 0.001).

Visually increased perilymphatic enhancement (PE) was present in 157 patients (87.7%) with unilateral EH (Figure 2). Quantitative BLB measurements confirmed a significant higher median SIR in affected ears of unilateral EH patients compared with their contralateral non-hydropic ears (*P* < 0.001; Table 4). In addition, there was a statistically significant trend of higher median SIR among increasing summative EH score (*T*_jt_ = 7.896,000; *z* = 4,104; *P* < 0.001; Figure 3). This trend was stronger for vestibular EH (*T*_jt_ = 5.640,000; *z* = 2,945; *P* = 0.003) than cochlear EH (*T*_jt_ = 1.943,000; *z* = 1,896; *P* = 0.058). Among the 22 patients with unilateral EH that did not demonstrate visually increased PE in their affected ear, 14 patients (63.6%) had isolated vestibular or isolated cochlear EH.

**Table 4:** Median SIR in hydropic and non-hydropic ears from 179 patients with unilateral HED.

	**Hydropic ears**	**Contralateral ears**	***P*-value**
Median SIR	1.40 [0.68–5.52]	1.05 [0.47–2.94]	<0.001

*SIR, signal intensity ratio; HED, Hydropic Ear Disease*.

Among the 15 bilateral EH patients with unilateral symptoms, the symptomatic ears demonstrated a mean SIR of 1.6 ± 0.8 compared with a mean SIR of 1.2 ± 0.5 in their symptomatic ear, which was statistically significant (*P* < 0.005). In addition, symptomatology in these patients was also correlated with a higher summative EH score [median EH score 1 (1–4) vs. 3 (1–5), *P* < 0.05].

### Correlation Between MRI Findings and Clinical Characteristics

#### Clinical Symptoms

Most patients reported concomitant vestibular and cochlear symptoms (212 patients, 98.6%). Three patients (1.4%) had isolated cochlear symptoms.

The clinical features from patients with unilateral EH are summarized in Table 5. There were no significant differences in the prevalence of hearing loss, tinnitus, or aural fullness in groups with different EH grades (*P* = 0.780; *P* = 0.900; *P* = 0.200). The median SIR also did not differ between hydropic ears with and without tinnitus or aural fullness (*P* = 0.947 and *P* = 0.185). Information on the frequency or duration of vertigo attacks was available in 102 (57.0%) and 166 (92.7%) patients with unilateral EH, respectively. No significant correlation was found between the degree of EH or SIR and the frequency of vertigo attacks (*P* = 0.188 and *P* = 0.165, respectively). Regarding the duration of vertigo, the majority of cases (84.9%) had vertigo episodes lasting between 20 min and 12 h. No significant differences were noted in summative EH score or median SIR across groups with different duration of vertigo attacks (*P* = 0.431 and *P* = 0.380, respectively).

**Table 5:** Clinical features in unilateral HED stratified by the summative EH score.

	**Grade 1**	**Grade 2**	**Grade 3**	**Grade 4**	**Grade 5**	***P*-value/*P*-value for trend**
Mean age, y	54.6 ± 2.2	58.8 ± 3.0	56.1 ± 1.9	54.5 ± 2.0	56.4 ± 1.7	0.732
Median disease duration, y	1.7 [0.1–30.0]	2.3 [0.2–30.0]	3.3 [0.04–24.8]	9.8 [0.25–33.0]	5.8 [0.33–29.0]	<0.001
Subjective hearing loss, *n* (%)	34 (94)	21 (100)	44 (98)	29 (97)	46 (98)	0.78
Vertigo, *n* (%)	34 (94)	21 (100)	45 (100)	29 (97)	47 (100)	0.2
Median nr of attacks per month	6.0 [0.3–28.0]	2.3 [0.3–14.0]	4.0 [0.08–70.0]	3.5 [1.0–28.0]	4.0 [0.16–16.0]	0.188
Duration of vertigo attacks						0.843
<20 min	1	1	2	1	2	
20 min−12 h	27	16	38	22	38	
12–24 h	1	1	0	1	0	
>24 h	1	1	1	0	0	
Variable duration	3	1	1	4	3	
Missing	3	1	3	2	4	
Tinnitus, *n* (%)	32 (89)	20 (95)	42 (93)	28 (93)	44 (94)	0.9
Aural fullness, *n* (%)	26 (72)	13 (62)	36 (80)	18 (60)	28 (60)	0.2
Drop attacks, *n* (%)	0 (0)	0 (0)	1 (0.02)	4 (13.3)	7 (14.9)	0.013

*HED, Hydropic Ear Disease; EH, endolymphatic hydrops*.

A comparison of clinical features between unilateral and bilateral EH patients is summarized in Table 6. No differences were found in the presence of aural symptoms (hearing loss, tinnitus, aural fullness) between patients with unilateral or bilateral EH. Additionally, no significant differences were noted in duration or frequency of vertigo attacks (*P* = 0.831 and *P* = 0.100, respectively).

**Table 6:** Clinical differences between unilateral and bilateral HED patients.

	**Unilateral EH**	**Bilateral EH**	***P*-value**
Mean age, *y*	55.9 ± 12.3	55.6 ± 14.2	0.870
Mean age of onset, *y*	48.13 ± 1.12	44.02 ± 3.08	0.145
Median disease duration, *y*	4.04 [0.04–33.0]	6.08 [0.3–45.9]	0.241
Hearing loss	*Hydropic ear*	*Worst ear*	
Median low Fletcher	48 [2–120]	53.5 [5–120]	0.190
Median high Fletcher	50 [2–120]	51 [5–120]	0.167
Median nr of vertigo attacks	4.0 [0.08–70]	2.0 [0.04–30.0]	0.100
per month			

*HED, Hydropic Ear Disease; EH, endolymphatic hydrops*.

#### Drop Attacks

Among patients with unilateral EH, drop attacks (DA) were reported by 12 subjects (Table 7). There were no statistically significant differences in sex or age between patients with and without DA (*P*= 0.236; *P* = 0.780). However, the DA group had a longer disease duration than patients without DA (*P* = 0.007). In all 12 patients with DA, MRI revealed concurrent cochlear and vestibular EH, which was both graded as either moderate or significant. The cochlear and vestibular EH grades, as well as the *summative* EH scores, were significantly higher in the DA group. Notably, DA was only reported in patients with a *summative* EH score of 3 or more. Additionally, patients with DA showed significantly greater hearing loss in the low and medium frequencies (low Fletcher 60 vs. 47, *P* = 0.027). No significant differences were noted at high frequencies between patients with and without DA (High Fletcher 58 vs. 48, *P* = 0.191).

**Table 7:** Characteristics of unilateral HED patients with and without drop attacks.

	**Drop attacks**	**No drop attacks**	***P*-value**
	***N* = 12**	***N* = 167**	
Male/female, *n* (%)	8/4 (67/33)	78/89 (47/53)	0.236
Mean age, *y*	55.0 ± 4.2	56.0 ± 0.9	0.780
Median disease duration, *y*	9.5 [3.2–23.2]	3.8 [0.04–33.0]	0.007
Median summative EH score	5.0 [3–5]	3.0 [1–5]	0.001
Median Cochlear EH score	2.0 [1–2]	1.0 [0–2]	0.013
Median Vestibular EH score	3.0 [2–3]	2.0 [0–3]	0.001
Median SIR	1.6 [0.83–3.66]	1.4 [0.68–5.52]	0.403
Median Low Fletcher	60 [45–72]	47 [2–120]	0.027
Median High Fletcher	58 [27–68]	48 [2–120]	0.191

*HED, Hydropic Ear Disease; SIR, signal intensity ratio; EH, endolymphatic hydrops*.

#### Comorbidities

Hypertension was the most frequent cardiovascular risk factor and present in 37 (17.2%) unilateral EH patients (18 men; 19 women), followed by dyslipidemia (9%) and smoking (7%). Twenty-four patients (11.2%) had a history of migraine. No significant differences in the prevalence of co-morbidities were noted between patients with unilateral or bilateral EH (data not shown).

#### Disease Duration

Information on disease duration was available in 142 unilateral HED patients (90.4%) and 27 bilateral HED patients (75%). In patients with unilateral EH, the Jonckheere-Terpstra test for trend revealed that the degree of EH increases significantly with longer disease duration (Table 5, *P* < 0.001 for the summative EH score; *P* < 0.001 for vestibular EH; *P* = 0.041 for cochlear EH). Among patients with a summative EH score of 5, the median disease duration was lower than would be expected. We performed an intra-cluster analysis of patients with a summative EH score of 5 with the aim of identifying factors associated with rapid EH progression. However, no correlation was found between disease duration and gender (*P* = 0.218), age (*P* = 0.137), vertigo attack frequency (*P* = 0.161), drop attacks (*P* = 0.705), SIR (*P* = 0.939), tinnitus (*P* = 0.081), aural fullness (*P* = 0.891), or sensorineural hearing loss (*P* = 0.823 for low Fletcher index, *P* = 0.886 for high Fletcher index).

Patients with bilateral EH did not demonstrate a statistically significant longer disease duration than patients with unilateral EH (6.1 yrs vs. 4.0 yrs; *P* = 0.241).

#### Pure Tone Audiometry

In 161 patients (89.9%) with unilateral EH and 36 patients with bilateral EH (100%), PTA was performed within 1 year of the MRI examination with a median time interval of 36 days (0–340 days). Of note, most patients (116/161, 72%) had undergone PTA within 2 months of the MRI examination.

In 161 unilateral EH patients, the average low Fletcher index was 48 dB [2; 120] in the hydropic ears and 13 dB [0–98] in the contralateral non-hydropic ears (*P* < 0.001). The high Fletcher index was 50 dB [2–120] in the hydropic ears and 17 dB [0–120] in the contralateral non-hydropic ears (*P* < 0.001). The Jonckheere-Terpstra test for trend showed that the low and high Fletcher indexes were significantly correlated with increasing summative EH score (*P* < 0.001 and *P* < 0.001; Table 8). A partial correlation was run to determine the relationship between SIR and hearing loss whilst controlling for EH. First, we performed a square root (sqrt) transformation of the low and high Fletcher indexes to reduce the skewness of our original data. The partial correlation revealed no statistically significant correlation between SIR and low Fletcher (*P* = 0.344) or high Fletcher (*P* = 0.178), respectively. We therefore did not demonstrate an additive effect of SIR on the extent of sensorineural hearing loss in HED patients.

**Table 8:** Hearing loss in unilateral HED stratified by the summative EH score.

	**Grade 1**	**Grade 2**	**Grade 3**	**Grade 4**	**Grade 5**	***P*-value for trend**
**Pure-tone audiometry**	***N* = 35**	***N* = 18**	***N* = 39**	***N* = 26**	***N* = 43**	
Median low Fletcher index	28.0 [2–120]	38 [20–75]	45 [12–120]	58 [5–75]	60 [22–85]	<0.001
Median high Fletcher index	26.5 [2–120]	42.0 [20–65]	51.0 [7–120]	60.0 [13–75]	60.0 [30–95]	<0.001
Missing	1	3	6	4	4	

*HED, Hydropic Ear Disease; EH, endolymphatic hydrops*.

The worst ear from bilateral EH patients demonstrated a median low Fletcher index of 53.5 dB [5–120] and median high Fletcher index of 51 dB [5–120]. These hearing thresholds were not significantly different from the low and high Fletcher indexes from the hydropic ears of unilateral EH patients (*P* = 0.190 and *P* = 0.167, respectively).

## Discussion

In the present study, we explored the relationship between cochleovestibular symptoms and intralabyrinthine abnormalities in a large cohort of patients with HED. We demonstrated the association between EH grade and hearing loss, as well as their progressive deterioration with respect to disease duration. Similar correlations have been shown by a couple of radiological studies (23, 27, 28, 30, 33) and a histological study on temporal bones from MD patients (47). Contrarily, previous radiological studies by Xie et al. (*N* = 117) and Fiorino et al. (*N* = 18) revealed no significant correlation between the severity of sensorineural hearing loss and EH grade, as revealed by MRI after intratympanic Gd-administration in definite MD patients (48, 49). Although the precise reason for this discrepancy is unknown, possible explanations may be different patient selection and sample sizes, or the use of a different MRI protocol and/or visual grading method for EH. Interestingly, our study revealed that there are cases with severe EH and hearing loss despite having a short disease duration and vice versa. These findings indicate that in some patients the hydropic process and hearing loss deteriorate rapidly, while others have a slow and gradual progression. Similar findings have previously been shown (50). Therefore, we hypothesize that factors other than duration are associated with the progression of EH. However, we did not identify factors associated with rapid EH progression. Further study is needed to explore these temporal patterns, which could have important implications in understanding the pathophysiology of HED.

Tinnitus and aural fullness have received less attention in literature, as emphasis is often placed on more prominent symptoms such as hearing loss and vertigo. However, it was previously reported that 19% of MD patients rank tinnitus as their most severe symptom and 38% considers aural fullness as a severe problem (51, 52). The identification of an *in vivo* correlate would provide important insights in the individually perceived severity of clinical disease. However, in contrast to hearing loss, our data did not demonstrate a correlation between EH grade and the presence of tinnitus or aural fullness. Notably, our study design did not cover qualitative measures of tinnitus or aural fullness, such as the perceived loudness or intensity.

The etiology of vertigo spells in HED also remains elusive. A myriad of theories have previously been proposed, including mechanical pressure or chemical imbalance/intoxication within the inner ear (53–56). Herein, we did not demonstrate a significant correlation between the extent of EH and the frequency or duration of vertigo attacks. It can therefore be concluded that merely the presence of EH does not elicit vertigo, which agrees with previous studies (23, 30). Schuknecht suggested that ruptures of the membranous labyrinth are the cause of vertigo spells, which would theoretically decrease the endolymph volume (57). However, a previous case report has revealed no large changes in EH on consecutive MRIs despite ongoing vertigo attacks (58). Of note, it was previously suggested that the fluctuations in EH may potentially be too small to be detected by MRI (23). Regarding the time course of vertigo attacks, our data contradicts previous suppositions that the number of attacks diminish over time (59). We did not observe a relationship between disease duration and the frequency or duration of vertigo attacks, which supports previous findings by Havia et al. and Jerin et al. (30, 60). It is worth noting that, although data on the *presence* of symptoms was well-documented in electronic patient files, we were unable to retrieve information on the *duration* of vestibular or cochlear symptoms in 21% of patients. Also, in hindsight, the categories we used to classify the duration of vertigo attacks were rather wide. The majority of patients fell within the 2nd category (vertigo attacks lasting 20 min to 12 h) and we did not account for further subanalysis.

In literature, there are conflicting views regarding the origin and evolution of EH. A previous meta-analysis of temporal bone reports from 184 MD patients demonstrated a cochleocentric distribution, where EH begins in the cochlear apex and from there progresses to involve the saccule, utricle and semicircular canals (61). The typical occurrence of low-frequency hearing loss in early stages of MD might be explained by these histopathological findings (57). On the contrary, in most of the radiologic literature, EH predominates in the vestibule (10, 62, 63). In our study, isolated cochlear EH was a scarce finding and less prevalent than isolated vestibular EH, which supports the hypothesis that EH originates in the vestibule. It was recently reported that a 3D real IR sequence is superior to 3D FLAIR in the assessment of cochlear EH due to its ability to distinguish endolymph from surrounding bone (64). Therefore, we cannot exclude that the lower prevalence of cochlear EH in our study cohort may be due to lower sensitivity of 3D FLAIR to cochlear EH. In addition, a golden standard for the assessment and quantification of EH is currently lacking. Although 3- and 4-point visual grading systems are often utilized to assess EH, and are subject to ongoing refinement, further developments are desired to enable objective volumetric quantification of EH.

Previous studies have addressed the development of bilateral EH in MD patients and reported variable prevalence rates ranging from 21 to 75%, which is thought to increase with disease duration (10, 23). In our study, bilateral EH was present in 36 (16.7%) of patients. We did not reveal significant differences in disease duration or age between patients with unilateral or bilateral EH and we were therefore unable to confirm the hypothesis. Nevertheless, the occurrence of bilaterality has led to the supposition that EH is a systemic disease and associations with genetic factors and several comorbidities have been reported in literature, particularly migraine and autoimmune diseases (21). In our study, migraine and autoimmune diseases were present in 11.2 and 6.5% of cases, respectively. Among the cardiovascular risk factors, hypertension was the most common comorbidity and reported in 17.2% of patients (17.8% in men; 16.7% in women). In comparison, the prevalence of arterial hypertension in the general Dutch population was estimated at 29.9% in men and 15.6% in women (65). Additionally, the prevalence of migraine in Europe and a broad group of autoimmune diseases in Denmark were estimated at 37.6 and 7.6–9.4%, respectively (66, 67). Therefore, an association between EH and autoimmune diseases, migraine or hypertension cannot be substantiated from our data.

The association between EH and BLB impairment at 4 h-delayed Gd-enhanced MRI has previously been demonstrated (32–36). In the present study, visually increased perilymphatic enhancement was present in 157 (87.7%) of unilateral HED patients. Furthermore, the degree of BLB impairment was significantly associated with EH grade. The BLB is indicated as the essential structure for the maintenance of inner ear fluid homeostasis by selectively regulating the passage of ions between the vasculature and inner ear (68). Based on this knowledge, it has been postulated that destruction of the labyrinthine barriers causes the formation of EH (33). However, previous studies have demonstrated that increased BLB permeability may also be present in non-hydropic ears from patients with sudden sensorineural hearing loss (SSNHL) or vestibular neuritis (36, 69). Therefore, it can also be deduced that BLB impairment does not necessarily lead to the formation of EH. Regardless of their actual pathophysiological processes, both EH and BLB impairment are likely markers of disordered labyrinthine homeostasis. Interestingly, it has been demonstrated on MRI that patients with SSNHL may show improvement of symptoms in parallel with resolution of the FLAIR hyperintensity (40). This close correspondence between MRI findings and clinical features in SSNHL patients raises the question whether this might also apply to patients with EH, though the underlying pathophysiological mechanisms are likely different. However, we evaluated the isolated effect of SIR on the degree of sensorineural hearing loss and did not demonstrate a significant correlation between SIR and low or high Fletcher indexes. Thus, a putative association between SIR and hearing loss in HED patients cannot be supported from this data. Our results are in agreement with Suzuki et al., who used the SIR of the basal cochlear turn against the cerebellar hemisphere as marker of BLB permeability and found no significant correlation with hearing thresholds (70). On the contrary, Kahn et al. did report a significant correlation between BLB impairment and worse hearing levels (71). In their study, however, they performed a visual assessment of BLB integrity and did not control for the potential confounding effect of EH. In addition to hearing loss, we also did not find a significant correlation between SIR and tinnitus, aural fullness, or the frequency and duration of vertigo attacks. It is worth noting that our retrospective study design inherently did not acknowledge certain clinical factors that could have potentially influenced the MRI results, such as the time elapsed between the MRI examination and the last vertigo attack or intratympanic corticosteroid injections. Longitudinal radiological and clinical assessment could offer a better characterization of the dynamic alteration of MRI parameters in relation to treatment strategies and symptomatology.

Drop attacks are known to occur in a subset of MD patients, although the reported incidence is variable with rates ranging from 13.5 to 72%, depending on the definition used (16, 24, 72). In our study, drop attacks were present in 7.4% of unilateral HED patients. We found that drop attacks were associated with a longer disease course and higher grades of EH compared to patients without DA's. The DA group also demonstrated greater hearing loss than non-DA patients, which may be attributed to the longer disease duration and greater degree of EH. Similar observations were previously made by Wu et al. (16). Contrarily, the median SIR was not significantly different between patients with and without DA. Although the pathophysiological mechanisms of drop attacks are unknown, mechanical stimulation of the otolithic organs (saccule and/or utricle) by endolymphatic pressure gradients is currently the most widely accepted etiology (73, 74). The finding that DA occurs in patients with a greater degree of EH seems to support this theory.

The rationale behind MR imaging of EH is based on a desire to seek improved diagnostic precision and better understand how labyrinthine abnormalities affect the function of the inner ear. In addition, the possibility that imaging-derived data could serve as a surrogate marker of disease progression and therapeutic efficacy has become attractive. A critical aspect when considering the use of an MRI as a surrogate parameter is validation: the degree of EH and/or BLB impairment must show a correlation with the presence and severity of audiovestibular symptoms. Investigating the clinical picture from a morphologic point of view (i.e., Hydropic Ear Disease) allows assessment of this pathologic stage across the entire clinical spectrum. Although some correlations have been found, the degree of EH and BLB impairment incompletely characterize the whole phenotype of HED, especially regarding the incidence and severity of vertigo attacks. If a more explicit radiologic-clinical correlation can be established, then this information could potentially be used to better predict disease progression, monitor treatment response, and aid the development of novel treatment strategies.

## Conclusion

In this retrospective study, we have investigated the clinical picture of patients with MRI-evidence of hydropic ear disease with or without BLB impairment. In our study population, the degree of EH correlates with disease duration, the severity of sensorineural hearing loss and the incidence of drop attacks. BLB is impaired in association with EH grade, but without clear contribution to the severity of audiovestibular symptoms.

## Data Availability Statement

The original contributions presented in the study are included in the article, further inquiries can be directed to the corresponding author/s.

## Ethics Statement

Ethical review and approval was not required for the study on human participants in accordance with the local legislation and institutional requirements. Written informed consent for participation was not required for this study in accordance with the national legislation and the institutional requirements.

## Author Contributions

LP, JS, and SH contributed to the conception and design of the manuscript. LP, SH, TV, MH, HB, and CB acquired the data. JG helped shape and verify the statistical methods. LP performed the statistical analysis and drafted the manuscript. All authors contributed to the article and approved the submitted version.

## Funding

Related to the research being submitted: SH: Radiology Research Fund from the Radiological Society of the Netherlands, to assess the technical feasibility of MRI-evaluated endolymphatic hydrops and allow development and optimization of imaging techniques. The funder was not involved in the study design, collection, analysis, interpretation of data, the writing of this article or the decision to submit it for publication. Not related to the research being submitted: BV: grants from Oticon and Advanced Bionics paid to institution.

## Conflict of Interest

The authors declare that the research was conducted in the absence of any commercial or financial relationships that could be construed as a potential conflict of interest.

## Publisher's Note

All claims expressed in this article are solely those of the authors and do not necessarily represent those of their affiliated organizations, or those of the publisher, the editors and the reviewers. Any product that may be evaluated in this article, or claim that may be made by its manufacturer, is not guaranteed or endorsed by the publisher.
